# Comparative evaluation of transforaminal lumbar interbody fusion and instrumented posterolateral fusion in Grade I/II Spondylolisthesis: A prospective study

**DOI:** 10.6026/973206300213426

**Published:** 2025-10-31

**Authors:** Abhiranjan Prasad, Narendra Kumar Das, Sourabh Guria, Diptiranjan Satapathy

**Affiliations:** 1 Department of Neurosurgery, KIMS, KIIT University, Bhubaneswar, India

**Keywords:** Spondylolisthesis, TLIF, PLF, spinal fusion, outcomes, comparative study

## Abstract

The optimal surgical approach between Transforaminal Lumbar Interbody Fusion (TLIF) and Posterolateral Fusion (PLF) for Grade I/II spondylolisthesis remains unclear. Therefore, it is of interest to compare clinical and radiological outcomes of TLIF versus PLF in patients with Grade I/II spondylolisthesis. PLF achieved better 6-month functional outcomes (ODI 12.95 ± 2.54 vs 17.83 ± 1.95, p<0.001) and greater slip reduction (48.45% vs 40.20%, p<0.001). TLIF provided superior early leg pain relief at 3 months (VAS 2.03 ± 0.80 vs 2.50 ± 0.91, p=0.015) and better DPQ scores. Complication and fusion rates were similar between groups. Both techniques are effective, with choice guided by patient profile and clinical priorities.

## Background:

Spondylolisthesis, derived from the Greek words "spondylos" (vertebra) and "olisthesis" (slippage), refers to the anterior displacement of one vertebral body relative to the one beneath it [1]. This condition affects approximately 6% of the adult population, with higher prevalence in certain demographic groups [2]. Among the various classification systems, the Meyerding classification remains the most widely used, categorizing spondylolisthesis into five grades based on the degree of vertebral slippage [3]. Grade I and II spondylolisthesis, representing up to 50% slippage, are the most frequently encountered in clinical practice and often associated with significant morbidity including low back pain, radiculopathy and neurogenic claudication [4]. Management of spondylolisthesis is normally with the use of conservative treatment which includes analgesics and physical activities. However, only a minor percentage of 20% of patients are forced to go through an operation due to persistent symptoms, progressive neurological impairments or instability of the spine [5]. The primary goals of surgery include a neural decompression, stabilization of the affected area and restoration of sagittal balance [6]. Transforaminal Lumbar interbody Fusion (TLIF) and Instrumented Posterolateral Fusion (PLF) have emerged as the surgeries of choice in low grade spondylolisthesis [7]. Unilateral transforaminal route of TLIF suggested by Harms and Rolinger in 1982 and popularized by Harms and Jeszenszky in 1998 is a procedure in which the surgeon interoughes the interbody fusion through a posterior approach [8]. Those theoretical advantages associated with this technique include restoration of disc heights, indirect decompression of the neural tissue and increased load-sharing by the anterior column [9]. On the other hand, the conventional approach to PLF that can be employed involves decortication of transverse processes and facet joints and then placement of bone grafts and pedicle screws without direct concerns with disc space [10].

Recent systematic reviews and meta-analyses have generated a conflicting result regarding the superior type of technique to another. Different surgical fusion techniques such as TLIF, PLIF, and PLF have been widely studied in spondylolisthesis. Rezk *et al.* reported that TLIF achieved superior functional outcomes and lower complication rates compared to PLIF in single-level, low-grade spondylolisthesis [11]. Similarly, Cheng *et al.* in a prospective controlled study, sought to compare clinical outcomes between PLIF and PLF techniques in spondylolisthesis cohorts [12]. A slightly more recent randomized controlled trial by Caelers *et al.* (2024) did not offer any meaningful differences in patient-reported outcomes between the two techniques at 12 months follow-up [13]. Despite these advances, several research gaps persist. Most comparative studies have focused primarily on short-term outcomes or fusion rates, with limited data on functional recovery patterns at different time points. Furthermore, the influence of surgical technique on quality of life measures and the relationship between radiological correction and clinical outcomes remain incompletely understood [14]. Additionally, there is a paucity of prospective studies directly comparing these techniques in well-matched patient populations with standardized assessment protocols. Therefore, it is of interest to comprehensively evaluate and compare the clinical outcomes, radiological parameters and complication rates between TLIF and PLF in patients with Grade I/II spondylolisthesis, with particular attention to functional recovery patterns at different postoperative time points.

## Materials and Methods:

This hospital-based prospective study was conducted at the Departments of Neurosurgery, Kalinga Institute of Medical Sciences and Pradyumna Bal Memorial Hospital, KIIT Deemed to be University in Bhubaneswar, Odisha, between February 2023 and May 2025. The study compared clinical and radiological outcomes of TLIF versus PLF in patients with Grade I/II spondylolisthesis.

## Study design and sample size:

A prospective comparative study design was employed with consecutive sampling of eligible patients. Based on a reported prevalence of spondylolisthesis of 6% [15], sample size was calculated using the formula:

Sample size = Z^2^1-α/2 x p(1-p)/d^2^

Where Z1-α/2 = 1.96 (for 95% confidence level), p = 0.06 (prevalence) and d = 0.08 (absolute error). This yielded a minimum sample size of 33.85, rounded to 40 patients per group for adequate statistical power.

## Inclusion and exclusion criteria:

## Inclusion criteria were:

[1] Adults aged 30-70 years of either sex

[2] Radiologically confirmed Grade I or II spondylolisthesis by X-ray and MRI

[3] Patients who provided informed consent for either TLIF or instrumented PLF

[4] Agreement to attend follow-up visits at 1, 3 and 6 months postoperatively

## Exclusion criteria were:

[1] Grade III or IV spondylolisthesis

[2] Lytic spondylolisthesis

[3] Pregnancy

[4] Psychiatric disorders

[5] Previous spinal surgery

[6] History of spinal fracture

## Surgical procedures:

All procedures were performed under general anesthesia with patients in the prone position. Single-level fusion was performed in each case.

## TLIF procedure:

A unilateral transforaminal approach was used. After pedicle screw placement, a facetectomy was performed to access the disc space. The disc was excised and an interbody cage filled with autograft or allograft was inserted. Rods were then connected to the pedicle screws for stabilization.

## PLF procedure:

A midline incision was made and paraspinal muscles were dissected to expose the posterior elements. After decortication of transverse processes and facet joints, bone graft was placed laterally. Pedicle screws were inserted bilaterally and connected with rods for stabilization.

## Assessment tools and Follow-up:

Patients were assessed at four time points: preoperatively and at 1, 3 and 6 months postoperatively. The following validated instruments were used:

[1] Visual Analog Scale (VAS): For assessment of low back pain and leg pain (0-10 scale)

[2] Oswestry Disability Index (ODI): For evaluation of functional disability (0-100%)

[3] Dallas Pain Questionnaire (DPQ): For assessment of pain impact on daily activities, work/leisure, anxiety/depression and social interest.

Operative variables including blood loss (ml), duration of surgery (minutes) and hospital stay (days) were recorded. At 6-month follow-up, radiological assessment was performed to evaluate slip reduction percentage and fusion status using the Bridwell grading system.

## Statistical analysis:

Data were analyzed using SPSS version 26.0. Continuous variables were presented as mean ± standard deviation (SD) and range. Categorical variables were expressed as frequencies and percentages. Independent t-tests were used for comparison of continuous variables between groups, while chi-square or Fisher's exact tests were employed for categorical variables. Repeated measures ANOVA were used to analyze changes in outcome measures over time. A p-value <0.05 was considered statistically significant.

## Results:

A total of 80 patients with Grade I/II spondylolisthesis were enrolled in the study, with 40 patients undergoing TLIF and 40 undergoing PLF. All patients completed the 6-month follow-up period, with no dropouts. The mean age of the study population was 50.13 ± 10.77 years (range 26-70 years). The TLIF group was significantly older than the PLF group (55.98 ± 9.68 years vs 44.27 ± 8.44 years, p<0.001). There were no significant differences between groups regarding gender distribution, comorbidities, level of lesion, or grade of slip (Table 1 - see PDF). Preoperatively, the TLIF group reported significantly higher pain scores for both low back pain (VAS: 7.73 ± 1.32 vs 7.10 ± 1.39, p=0.043) and leg pain (VAS: 5.90 ± 1.28 vs 5.25 ± 1.14, p=0.028). No significant differences were observed in ODI (66.63 ± 9.57 vs 66.87 ± 10.18, p=0.910) or DPQ scores (45.13 ± 8.89 vs 45.65 ± 8.68, p=0.790) between groups (Table 2 - see PDF). The TLIF procedure was associated with significantly greater blood loss (103.25 ± 25.66 ml vs 90.25 ± 16.94 ml, p=0.009), longer operative duration (121.25 ± 17.16 minutes vs 112.00 ± 14.62 minutes, p=0.011) and longer hospital stay (7.02 ± 1.35 days vs 6.00 ± 1.24 days, p=0.001) compared to PLF (Table 2 - see PDF). At 1-month follow-up, no significant differences were observed between groups in VAS scores for low back pain (4.57 ± 1.26 vs 4.65 ± 0.92, p=0.762) or leg pain (3.50 ± 0.93 vs 3.63 ± 0.93, p=0.549), ODI scores (35.28 ± 7.92 vs 36.23 ± 6.92, p=0.569), or DPQ scores (25.70 ± 5.98 vs 26.07 ± 5.13, p=0.764) (Table 2 - see PDF). At 3-month follow-up, the TLIF group showed significantly lower VAS scores for leg pain (2.03 ± 0.80 vs 2.50 ± 0.91, p=0.015) and better DPQ scores (21.18 ± 1.97 vs 22.07 ± 1.37, p=0.020). No significant differences were observed in VAS scores for low back pain (3.20 ± 0.79 vs 3.50 ± 0.88, p=0.112) or ODI scores (22.23 ± 1.75 vs 22.50 ± 1.26, p=0.422) (Table 2 - see PDF). At 6-month follow-up, the PLF group demonstrated significantly better functional outcomes as measured by ODI (12.95 ± 2.54 vs 17.83 ± 1.95, p<0.001). No significant differences were observed in VAS scores for low back pain (2.00 ± 0.78 vs 2.30 ± 0.69, p=0.073), leg pain (1.48 ± 0.60 vs 1.63 ± 0.71, p=0.308), or DPQ scores (18.13 ± 1.16 vs 18.40 ± 1.24, p=0.308) (Table 2 - see PDF). At 6-month follow-up, PLF demonstrated significantly greater slip reduction compared to TLIF (48.45 ± 2.60% vs 40.20 ± 3.11%, p<0.001). Fusion rates were 55% in the TLIF group and 70% in the PLF group, though this difference was not statistically significant (p=0.166). No significant differences were observed in complication rates between groups (p=0.863), with dural tear (7.5% vs 10%), nerve root injury (7.5% vs 5%), wound infection (5% vs 10%) and other complications (10% vs 12.5%) occurring in similar proportions (Table 3 - see PDF).

## Discussion:

This future comparative study provides substantive evidence concerning clinical and radiological outcomes of the usage of thoracolumbar interbody fusion (TLIF) and posterior lumbar fusion (PLF) in spondylolisthesis patients with Grade I/II. The findings show clear superiority of each method at different intervals of postoperative time, PLF has stronger functional outcomes and radiological correction after six months of time and TLIF has strong early analgesia after three months. It is worth noting that the comparative outcomes on functional outcomes by the PLF cohort at six months were significantly better as indicated by Oswestry Disability Index (ODI) of 12.95 (2.54) versus 17.83 (1.95), which is contrary to some previous studies. As an example, a 2020 meta-analysis by Kalichman *et al.* found no significant differences between TLIF and PLF in the 12-month follow-up of functional outcomes [15]. Our findings are consistent with the meta-analysis by Phan *et al.* which showed that minimally invasive TLIF can achieve comparable clinical and fusion outcomes to open TLIF with reduced perioperative morbidity [16]. The better functional results of the PLF group could be explained by many factors such as the shortened length of the operation, minimized intraoperative bleeding and shorter hospitalization, which revealed significant differences in our cohort. These factors can be combined to promote faster recovery and promptly promote a quick recovery to usual activities thus leading to increased functional performance after six months [17]. The significantly greater slip reduction achieved with PLF (48.45±2.60% vs 40.20±3.11%, p<0.001) is another important finding. This contrasts with the theoretical advantage of TLIF in restoring sagittal alignment through interbody support. However, our results are consistent with a 2021 study by Farrokhi *et al.* which also reported better slip reduction with PLF, possibly due to the ability to perform more aggressive reduction without the constraints of interbody space preparation [18]. The clinical significance of greater slip reduction remains debated, as our study did not find a direct correlation between the degree of slip reduction and pain outcomes or functional improvement. This suggests that while radiological correction is an important surgical goal, it may not necessarily translate to better clinical outcomes in all cases. The superior leg pain reduction observed with TLIF at 3 months (2.03±0.80 vs 2.50±0.91, p=0.015) and better DPQ scores at the same time point (21.18±1.97 vs 22.07±1.37, p=0.020) highlight the potential advantages of TLIF in the early postoperative period. These findings support the theoretical benefits of TLIF in providing indirect decompression of neural elements through disc height restoration and foraminal widening [19]. Pooswamy *et al.* (2017) compared TLIF and instrumented PLF in Grade I/II spondylolisthesis and found that both techniques yielded significant postoperative improvement, with TLIF showing better leg pain relief and PLF demonstrating slightly superior radiological correction at mid-term follow-up [20]. Other studies similarly reported better early pain relief with TLIF, attributing this to more effective decompression of neural elements [21]. However, by 6 months, these differences were no longer significant, suggesting that while TLIF may provide faster pain relief both techniques ultimately achieve similar pain control outcomes.

The lack of significant differences in fusion rates at 6 months (55% vs 70%, p=0.166) is noteworthy, as previous studies have suggested higher fusion rates with TLIF due to the larger surface area available for bone grafting in the interbody space [22]. However, our findings align with a 2023 systematic review by Yan *et al.* which concluded that fusion rates between TLIF and PLF become comparable with longer follow-up periods [23]. The relatively short follow-up period in our study may have contributed to these findings, as fusion typically takes 9-12 months to mature fully. Longer-term studies are needed to definitively compare fusion rates between these techniques. The comparable complication rates between groups (p=0.863) are reassuring and consistent with previous studies [24]. The slightly higher rate of wound infection in the PLF group (10% vs 5%) may be attributed to the more extensive muscle dissection required in this approach, though this difference was not statistically significant. The similar rates of neurological complications (dural tear and nerve root injury) suggest that both techniques can be performed safely with appropriate expertise. Zhang *et al.* (2017) observed that minimally invasive TLIF achieved comparable clinical and radiological outcomes to PLIF, with reduced operative time and perioperative morbidity, supporting the notion that technique selection should be individualized to patient factors and surgeon expertise [25]. There are several limitations of this study that need to be enumerated. One, comparatively-long follow-up of 66 months may not capture long-term outcomes such as subsequent degeneration of adjacent segments or subsequent complications. Second, the applicability of the results to other locations may be impaired by the single-center design. Third, some selection bias could have been introduced by the lack of randomization, but the matched groups were demographic and clinical feature-matched. Finally, the differences on rare complications or subgroup analysis in relation to level of lesion or grade of slip were not identified by power analysis. However, this study provides practical comparative data of two popular surgical procedures on Grade I/II spondylolisthesis. These findings suggest that both TLIF and PLF are effective surgery methods and that they have specific advantages at different time intervals. PLF appears to achieve better functional outcome and radiological correction after 6 months, but TLIF better early analgesia. The results suggest an individualized character of surgical decision-making, to involve the patient factors, clinical priorities and surgeon experience.

## Conclusion:

This comparative analysis shows that TLIF and PLF are viable surgery techniques in Grade I/II spondylolisthesis with certain advantages at different postoperative periods. The patients treated with PFL reported superior functional performance at 6 months and lesser more slip and TLIF provided superior early pain relief at 3 months. Complication rates and fusion rates differed significantly at 6month.

## References

[1] Wiltse LL (1976). Clin Orthop Relat Res..

[2] Jacobsen S (2007). Spine (Phila Pa 1976)..

[3] Koslosky E, Gendelberg D (2020). Clin Orthop Relat Res..

[4] Matsunaga S (1990). Spine (Phila Pa 1976)..

[5] Herkowitz HN, Kurz LT. (1991). J Bone Joint Surg Am..

[6] Ghogawala Z (2016). N Engl J Med..

[7] Resnick DK (2005). J Neurosurg Spine..

[8] Harms J, Rolinger H (1982). Z Orthop Ihre Grenzgeb..

[9] Lowe TG, Tahernia AD (2002). Clin Orthop Relat Res..

[10] Christensen FB (2004). Acta Orthop Scand Suppl..

[11] Ali Rezk EM (2019). Egypt J Neurosurg..

[12] Cheng L (2008). Int Orthop..

[13] Caelers IJ (2024). Lancet Reg Health Eur..

[14] Hartmann S (2022). Neurosurg Rev..

[15] Kalichman L (2009). Spine (Phila Pa 1976)..

[16] Phan K (2015). Eur Spine J..

[17] Said E (2021). Global Spine J..

[18] Farrokhi MR (2021). Indian J Neurotrauma..

[19] Kono Y (2018). Asian Spine J..

[20] Pooswamy S (2017). Indian J Orthop..

[21] de Kunder SL (2017). Spine J..

[22] Kim MC (2011). Asian Spine J..

[23] Yan DL (2008). Eur Spine J..

[24] Kim CH (2013). Spine (Phila Pa 1976)..

[25] Zhang D (2017). Medicine (Baltimore)..

